# Patterns and drivers of species richness and turnover of neo-endemic and palaeo-endemic vascular plants in a Mediterranean hotspot: the case of Crete, Greece

**DOI:** 10.1186/s40709-019-0106-x

**Published:** 2019-11-05

**Authors:** Maria Lazarina, Athanasios S. Kallimanis, Panayotis Dimopoulos, Maria Psaralexi, Danai-Eleni Michailidou, Stefanos P. Sgardelis

**Affiliations:** 1 Department of Ecology, School of Biology, Aristotle University of Thessaloniki, 54124 Thessaloniki, Greece; 2 Department of Biology, Laboratory of Botany, University of Patras, Rio, 26504 Patra, Greece

**Keywords:** Endemism, Zeta diversity, Spatial turnover, Multi-site Generalized Dissimilarity Modelling, Beta diversity

## Abstract

**Background:**

Exploring species richness and turnover patterns and their drivers can provide new insights into underlying mechanisms shaping community assembly, with significant implications for biodiversity conservation. Here, we explored diversity patterns of non-endemic, neo-endemic and palaeo-endemic vascular plants in Crete, Greece, a Mediterranean hotspot of plant richness and endemism. We evaluated the relationship between α-diversity and environmental (bioclimatic variables, topography), and anthropogenic variables by Generalized Additive Models, after accounting for spatial autocorrelation. Then, we quantified turnover using the novel concept of zeta diversity (the number of shared species by multiple sites), a framework which allows to explore the full spectrum of compositional turnover, the contribution of rare and widespread species to observed patterns and the underlying processes shaping them. Finally, we explored the abiotic and biotic effects, i.e. how well one category of species (non-endemics, palaeo-endemics, neo-endemics) predicts the patterns of the other categories, on zeta diversity by multi-site Generalized Dissimilarity Modelling.

**Results:**

We found a strong correlation between neo-endemic and palaeo-endemic α-diversity, with climate, topography, and human impact driving species richness. Zeta diversity analysis revealed a sharper decrease of shared palaeo-endemic species, followed by neo-endemics, and then by non-endemics with the number of sites considered to estimate compositional turnover. Perhaps, the narrow distributions of palaeo-endemics as relict species and often habitat specialists, thus persisting locally, and of neo-endemics that may have not reached yet their potential geographical range, resulted in the observed zeta diversity decline pattern. Deterministic processes controlled species turnover of rare non-endemic and neo-endemic species, while deterministic and stochastic processes contributed similarly to palaeo-endemic turnover. However, stochasticity dominates in the case of widespread species in all occasions. The environmental and anthropogenic variables were poor predictors of compositional turnover, especially of widespread species. However, the non-endemic species composition was correlated to rare palaeo-endemics and neo-endemics, highlighting the importance of biotic effects in driving turnover patterns.

**Conclusions:**

It seems that centers of neo-endemism of vascular plants coincide with centers of palaeo-endemism in Crete, but species richness and species turnover are shaped by different drivers.

## Background

The understanding of the diversity patterns along spatial scales provides invaluable insights into species distribution and underlying assembly processes [1–3] with significant implications for biodiversity conservation [4]. Whittaker [5, 6] proposed the partitioning of diversity into three components: α-, β-, and γ-diversity defined as the species richness at local scale, the variation in species composition, and species richness at regional scale, respectively. Patterns of species richness across spatial scales have been widely studied [7–9], whereas there is a growing research interest for β-diversity patterns. β-diversity reflects how communities respond to environmental gradients and changes, and climate change [4, 10–13]. Among the most often used metrics of β-diversity are pairwise (dis)similarity indices (e.g. Jaccard index) quantifying changes (or similarity) in species composition between a pair of sites [14, 15]. However, pairwise β-diversity metrics do not quantify compositional differences across more than two sites failing to fully describe turnover patterns, while they are sensitive to rare species with widespread species contributing less to turnover [16–18].

The novel concept of zeta (ζ) diversity defined as the number of species shared by multiple sites was proposed by Hui and McGeogh [19] to resolve these issues. Zeta diversity by quantifying the overlap of species distributions across multiple sites, overcomes the limitation of the pairwise comparisons of many widely used β-diversity metrics, reflecting the full spectrum of multi-site compositional turnover patterns [19]. Therefore, it offers a framework that links diversity patterns across spatial scales, with this link representing a crucial desideratum in the biodiversity conservation [4]. Furthermore, zeta diversity allows us to evaluate the contribution of rare, intermediate-ranging and widespread species to turnover patterns [17]. This is an important property, since although species rarity and the associated extinction risk is commonly used for prioritization of species and conservation planning [20, 21], not only rare, but also common species, the ones “that shape the world around us” [22] contributing substantially to the ecosystem functioning [23], are calling for conservation [24]. To sum up, zeta diversity as it is linked to all facets of diversity [25], although is comparatively still in its infancy, has the potential to provide in-depth insights into the turnover patterns and the underlying community assembly processes driving them [18, 26–29], the species-area relationship [30] and the scaling of endemism [19].

In the Mediterranean region, the geological and historical events created a unique biodiversity hotspot where approximately 10% of the world’s higher plants are found, with an astounding number of endemic species [31, 32]. Within the region, the island of Crete is considered a plant richness and endemism hotspot [31]. The endemic flora of Crete is composed of relict species of a past flora (palaeo-endemics sensu Stebbins and Major [33]) and recently evolved species (neo-endemics sensu Stebbins and Major [33]) [34]. Cretan relict flora consists of lowland species, while diversification processes occurred at mountains [35]. Palaeo-endemics have suffered range contraction due to past climatic changes (e.g. during the Pleistocene) and are restricted to a fraction of their original distribution, often persisting in refugia (e.g. cliffs in the Mediterranean area). They are considered taxonomically isolated taxa including usually mono- or oligo-typic genera. On the other hand, neo-endemics are taxa that have evolved recently, include polytypic genera, and might have not reached yet their potential distribution due to their young age. In this context, palaeo-endemics and neo-endemics are linked to “museums” and “cradles” of biological diversity i.e. speciation centers [32, 36], and therefore understanding their diversity patterns and drivers shaping them will provide useful input for biodiversity conservation.

In the present study, we explored the patterns and drivers of species richness and turnover of vascular plant species in a Mediterranean hotspot considered as center of endemism [31, 37, 38], the island of Crete, Greece. We performed the investigation for three species categories: non-endemic, palaeo-endemic and neo-endemic species. We quantified turnover with zeta diversity, a suitable approach for comparing different species groups’ turnover within the same study area [25], to capture the full range of compositional turnover and the contribution of species ranging from rare to widespread of each category. First, we explore if the species of different categories differ in their elevational and geographical range. Next, we ask if centers of palaeo-endemism, neo-endemism and of non-endemic species richness coincide, and which climatic and anthropogenic factors affect the species richness of different categories. Finally, we explore the turnover and the co-occurrence patterns quantified by zeta diversity of non-endemic, palaeo-endemic and neo-endemic species, and the drivers and the underlying processes shaping the observed patterns. We expect that as palaeo-endemics are narrow-ranging species that evolved in past climatic conditions and are perhaps specialists persisting only locally will exhibit a sharper decline in multi-site compositional turnover possibly driven by deterministic processes. Contrarily, given the reported reduced turnover of island floras due to the wide distribution of non-endemic species driven by both stochastic and deterministic processes [39], a less steep decline is expected for the non-endemic group. Finally, we expect that neo-endemics will lie between two extremes.

## Methods

### Study area

Crete, the largest island of Greece, is located in the southern part of the Aegean Sea. The climate is Mediterranean with long hot and dry summers and mild winters, with mean annual temperature ranging from 9.94 to 19.13 °C and mean annual precipitation from 486.50 to 1035.32 mm (data from WorldClim [40]). The area is characterized by mountainous terrain with three mountain massifs: Lefka Ori (2452 m, west Crete), Psiloritis (2456 m, central Crete) and Dikti (2148 m, east Crete). Biogeographically, the island belongs to the floristic region of Kriti and Karpathos (including satellite islands = the Cretan area) which comprises 2079 species and 571 subspecies [41]. The Cretan vascular flora includes 1647 species and subspecies (hereafter species) with approximately 10% of them being endemic to Crete [42] and 17.6% of them being endemic to Greece [32, 43].

### Data

We used floristic data obtained by the distribution maps (grid size: 8.25 × 8.25 km, 160 grid cells, hereafter sites) of individual plant species from Crete provided by the “*Flora of the Cretan area: annotated checklist and atlas*” of Turland et al. [42]. Species were classified into three categories: non-endemics (NON-E), single-island neo-endemics (NE), and single-island palaeo-endemics (PE) using data provided in Kallimanis et al. [44] and any subsequent analysis was performed separately for each species category. Briefly, Kallimanis et al. classified species into different categories using published studies for specific taxa (e.g. Cellinese and Smith [45] for endemic Campanulaceae of Crete and Greuter [46] about the relict Cretan flora) and a criterion of systematic isolation, congruent with Stebbins and Major [33] definition of palaeo- and neo-endemism. Therefore, as palaeo-endemics were assigned isolated (reproductively and geographically) species with no close relatives and as neo-endemics all taxa at subspecific rank including vicariant species. We acknowledge that literature and systematic based formulation of endemic species categories—due to lack of phylogenetic information e.g. the public database TimeTree [47] included only 34 out of the 165 species [a short description of the performed analysis to generate timed phylogeny is presented in Supplementary along with the tree generated by TimeTree (Additional file 1: Figure S1)]—is certainly a limitation of our study.

For each site, we obtained topographical data (elevation, aspect, and slope) from a digital surface model produced in the framework of the Reference Data Access (RDA) Action of the EU GMES/Copernicus program (Copernicus land monitoring services 2018). The climate was quantified by the 19 bioclimatic variables of the WorldClim dataset [40]. Specifically, we aggregated grid cell values from the high-resolution WorldClim dataset (30 arc-seconds, ~ 1 km) using zonal statistics in ArcGIS 10.3 to calculate mean variable values per site. Furthermore, to quantify human effect, we calculated the percentage of human land uses (i.e. artificial surfaces, arable land, permanent crops, pastures, and heterogeneous agricultural areas) using the CORINE Land Cover 2000 database (Copernicus land monitoring services 2018) and mean human population density per site. The calculations were performed in ArcGIS 10.3 (ArcGIS^®^ software by ESRI).

### Statistical analysis

#### Elevational range and species distribution

We explored if species belonging to different categories demonstrated different elevational and geographical ranges. To this end, we estimated (a) the mean elevation of each species’ occurrence, (b) the range i.e. minimum–maximum elevation of occurrence, and (c) the ratio of the number of sites each species occupies to the total number of sites. Then, we evaluated differences among categories by performing permutational one-way ANOVA with the function *aovp* of the R package *lmPerm* [48]. In the case of significant differences, we implemented Tukey HSD post-hoc tests for pairwise comparisons. Furthermore, we estimated the C-score and the NODF index to quantify species co-occurrence and identify patterns of nestedness in species composition respectively with the *bipartite* package [49].

#### α-diversity

We estimated α-diversity i.e. species richness at site level for each species category, and we explored the relationship between species richness of different categories per two by Generalized Additive Models (GAM) with Poisson error distribution and log-link function (thin plate regression splines and 3 knots per spline). Furthermore, we evaluated the effect of bioclimatic variables, topography, and human effect on α-diversity of each species category with GAMs. To account for spatial autocorrelation we included the distance-based Moran’s eigenvectors with positive values that were estimated by the *adespatial* package [50] to the GAM. Prior to analysis, we performed Variance Inflation Factor (VIF) analysis to test for multicollinearity between bioclimatic variables, topographical variables and human effect related variables with the function *vifstep* of the *usdm* package [51], and setting VIF criterion < 10. The analysis indicated multicollinearity and the variables satisfying the criterion and used in the analysis were: isothermality, temperature seasonality, precipitation seasonality, precipitation of the wettest quarter of the year, precipitation of the warmest quarter of the year, aspect, slope, human population density, and the percentage of human land uses.

#### Species turnover

We estimated the zeta diversity decline and the ratio of zeta diversity decline for NON-E, NE and PE vascular plants with the package *zetadiv* [52]. Briefly, zeta diversity is defined as the mean number of shared species in *n* number of sites [19]; the number of sites that is used to estimate zeta diversity and is referred as zeta order (hereafter ζ_i_ for *i* different number of sites). For zeta order of 1, the zeta diversity corresponds to the mean α-diversity i.e. mean species richness per site (ζ_1_), while for order 2 is the mean number of shared species in two sites (ζ_2_). Therefore, all the incidence-based pairwise β-diversity metrics can be estimated using ζ_1_ and ζ_2_. Zeta diversity for zeta orders from 3 to *n* is computed as the number of species in common in three to the total number of sites, with higher orders of zeta reflecting the contribution of more widespread species to compositional change and lower orders providing information on the rare species. Given that the shared species of *n* sites are necessarily shared in the *n* − 1 sites and the number of shared species declines as more sites are considered to calculate number of shared species, zeta diversity declines with zeta order. The zeta diversity decline is usually described by power-law or negative exponential function and it is informative about underlying processes driving differentiation of species composition, and the role of common and rare species in species turnover patterns [17]. Specifically, a power-law decline, i.e. species have a unique chance to occur in a site, is indicative that niche-based processes drive species turnover, while an exponential decline i.e. species have an equal chance to occur in a site, implies that stochasticity overrules. The zeta ratio ζ_i_/ζ_i+1_ termed as the retention rate, quantifies the proportion of species which are retained with the addition of more sites to estimate zeta diversity. The relationship between zeta ratio and zeta order (zeta order here corresponds to the denominator *i *+ 1) inform us about the probability of a species to be retained as more sites are considered in the computation, hence for the rarity and commonness of species [25]. Here, following Latombe et al. [27], we estimated zeta decline and retention rate for each species category and estimated the parametric form of the former relationship piece-wisely to detect different patterns between widespread and rare species. Additionally, we estimated the β-diversity index N* [53] for comparison.

We applied the framework of Multi-Site Generalized Dissimilarity Modelling (MS-GDM) [17] to explore the effect of environmental (bioclimatic, topographic) and anthropogenic variables transformed with I-splines on zeta diversity (abiotic model) with the package *zetadiv* [52] and the functions provided by Latombe et al. [27]. The importance of the predictor variable is evaluated by the maximum value of the spline, and the variation in slope across splines shows at which range of the predictor variable, the latter exerts more important effect on the differences of species composition [17]. We quantified zeta diversity by Sørensen and Simpson version of zeta diversity. The indices are rescaled to 0–1, by dividing zeta diversity with mean α-diversity and the minimum richness of sites for the Sørensen and Simspon index, respectively. The scheme is analogous to Baselga’s partitioning of β-diversity into nestedness and turnover components [54], with the Sørensen version of zeta diversity reflecting nestedness component thus richness-dependent turnover and Simpson reflecting “true” turnover. Furthermore, we explored the effect of NON-E composition on PE and NE composition by adding the zeta diversity of ΝΟΝ-Ε as independent variable to the MS-GDM of NE and PE (biotic model I). Finally, we tested how the NE composition affected PE composition and vice versa (biotic model II). For each MS-GDM we estimated the variance explained as the Pearson R^2^ obtained by the relationship between the observed and predicted z values.

All analyses were performed in R version 3.5.2 (R Development Core Team, 2018).

## Results

### Elevational range and species distribution

Palaeo-endemics and neo-endemics exhibited significant differences in mean elevation and minimum elevation of occurrence (Table 1), with non-endemics (NON-E) showing higher values, followed by neo-endemics (NE). NON-E had significantly wider distributions than palaeo-endemics (PE), and NE exhibited greater, but no significant, geographical range than PE. The lowest value of C-score and the lowest value of NODF were estimated for PE.

**Table 1:** Elevational range (mean values ± standard deviation of mean, minimum, and maximum values), geographical range (mean values ± standard deviation), diversity indices (γ-diversity, mean α-diversity, and β-diversity estimated by N* index), along with index NODF and C-score reflecting nestedness and species co-occurrence of non-endemic, neo-endemic and palaeo-endemic vascular plants in Crete, Greece

	Non-endemics	Neo-endemics	Palaeo-endemics
Elevation			
Mean	341.88 ± 162.14^a^	434.34 ± 182.06^b^	381.92 ± 157.12^a^
Minimum	128.02 ± 167.36^a^	204.83 ± 206.35^b^	161.99 ± 146.77^a^
Maximum	*776.10 *± *397.77*	*839.13 *± *459.80*	*726.88 *± *377.90*
Range	*648.09 *± *509.98*	*634.30 *± *466.55*	*564.89 *± *412.59*
Geographical range	0.08 ± 0.09^a^	0.06 ± 0.06^b^	0.05 ± 0.05^b^
Diversity			
γ-diversity	1482.00	91.00	74.00
Mean α-diversity	115.04	5.18	3.63
β-diversity index N*	12.10	18.58	22.03
NODF	20.73	25.48	27.97
C score	0.76	0.70	0.63

Different letters indicate significant differences in elevational and geographical range between different species categories, according to permutational one-way ANOVA, while non-significant differences are indicated in italics

### α-diversity

Among the 1647 species, 1482 were NON-E, 91 NE, and 74 PE. There were no significant differences in α-diversity between PE and NE (permutational ANOVA), but there were more NE than PE species in approximately 60% of the sites (Additional file 1: Figure S2). We observed significant positive relationships between NON-E and NE, and NE and PE (R^2^ = 0.24, deviance explained = 30.00%, and R^2^ = 0.80, deviance explained = 74.20% respectively, *p* < 0.05), but the relationship between NON-E and PE was hump-shaped (R^2^ = 0.13, deviance explained = 22.50%, *p* < 0.05) with declining PE species richness when NON-E species richness was high (Additional file 1: Figure S3). Furthermore, we found that all the bioclimatic, topographical, and human effect variables had a significant effect on NON-E α-diversity, after accounting for spatial autocorrelation (R^2^ = 0.18, deviance explained = 36.30%, *p* < 0.05, Additional file 1: Figure S4). NE α-diversity was significantly correlated to precipitation seasonality, slope and human population density (R^2^ = 0.26, deviance explained = 43.50%, *p* < 0.05, Additional file 1: Figure S5). Finally, temperature seasonality, precipitation of the wettest and warmest quarter and human population density had a significant effect on PE α-diversity (R^2^ = 0.48, deviance explained = 55.80%, *p* < 0.05, Additional file 1: Figure S6).

### Species turnover

**Fig. 1:**
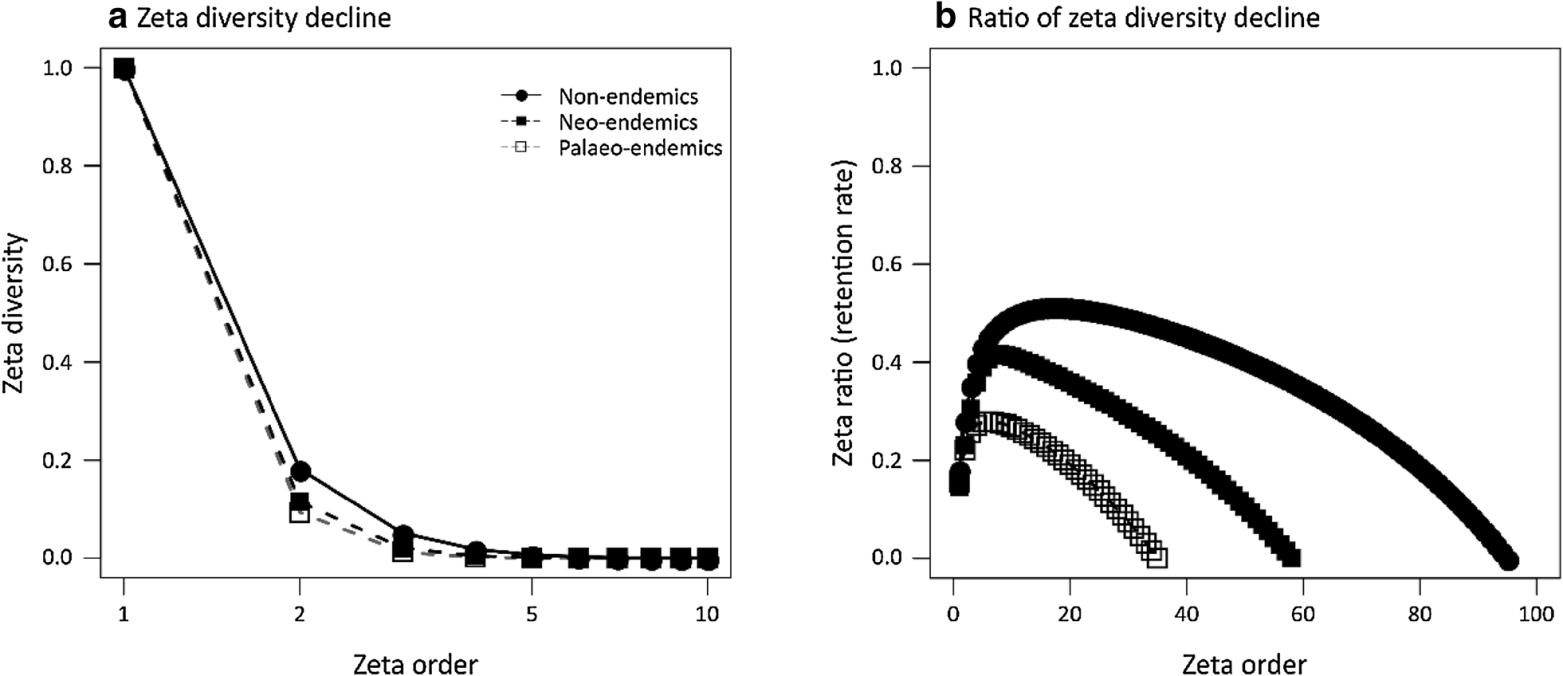
The zeta diversity decline rescaled to 0–1 (**a**) and ratio of zeta diversity decline (**b**) with zeta order for non-endemics, neo-endemics and palaeo-endemics vascular plants of Crete, Greece

Palaeo-endemics exhibited the highest β-diversity according to N* index, followed by neo-endemic. The decline of zeta diversity (rescaled to 0–1) with zeta order was slightly sharper for PE, followed by NE (Fig. 1a). Therefore, the retention rate was lower for PE. This means that the number of shared PE species declined sharper as more sites were included for the zeta diversity estimation. The relationship of the zeta ratio with order showed that PE and NE retention rate increased sharply up to 6 and 8 zeta order respectively, and then decreased, with the decreasing part being sharper for PE (Fig. 1b). On the other hand, the retention rate of NON-E species increased with order initially and then reached a rough plateau to decrease at higher zeta orders. Based on the shape of the retention rate relationship with zeta order, we formulated two categories of commonness for each species category, differentiating species into rare (low zeta orders corresponding to the increasing part of the retention rate) and widespread (higher zeta orders corresponding to the decreasing part of the retention rate), and estimated the parametric form piece-wisely of the zeta decline for rare and widespread. The exploration revealed that the zeta decline was described by a combination of negative exponential and power-law functions for rare species, while the decline was exponential for the widespread species (Table 2). The PE exhibited similar exponential and power-law coefficient for rare species, whereas for rare NON-E and NE the power-law coefficient was greater (Table 2).

**Table 2:** The estimated coefficients and the significance of the fit of the exponential and power-law of the zeta decline for non-endemic, neo-endemic, and palaeo-endemic of vascular plants of Crete, Greece

Species category	Exponential coefficient	Power-law coefficient
Non-endemics		
Zeta Order ≤ 17	0.55 (*p *< 0.001)	1.68 (*p* < 0.001)
Zeta Order > 17	1.11 (*p* < 0.001)	0 (*p* = 1)
Neo-endemics		
Zeta Order ≤ 8	0.59 (*p* < 0.001)	2.01 (*p* < 0.001)
Zeta Order > 8	1.43 (*p* < 0.001)	0 (*p* = 1)
Palaeo-endemics		
Zeta Order ≤ 6	1.04 (*p* < 0.001)	1.16 (*p* < 0.001)
Zeta Order > 6	1.82 (*p* < 0.001)	0 (*p* = 1)

**Fig. 2:**
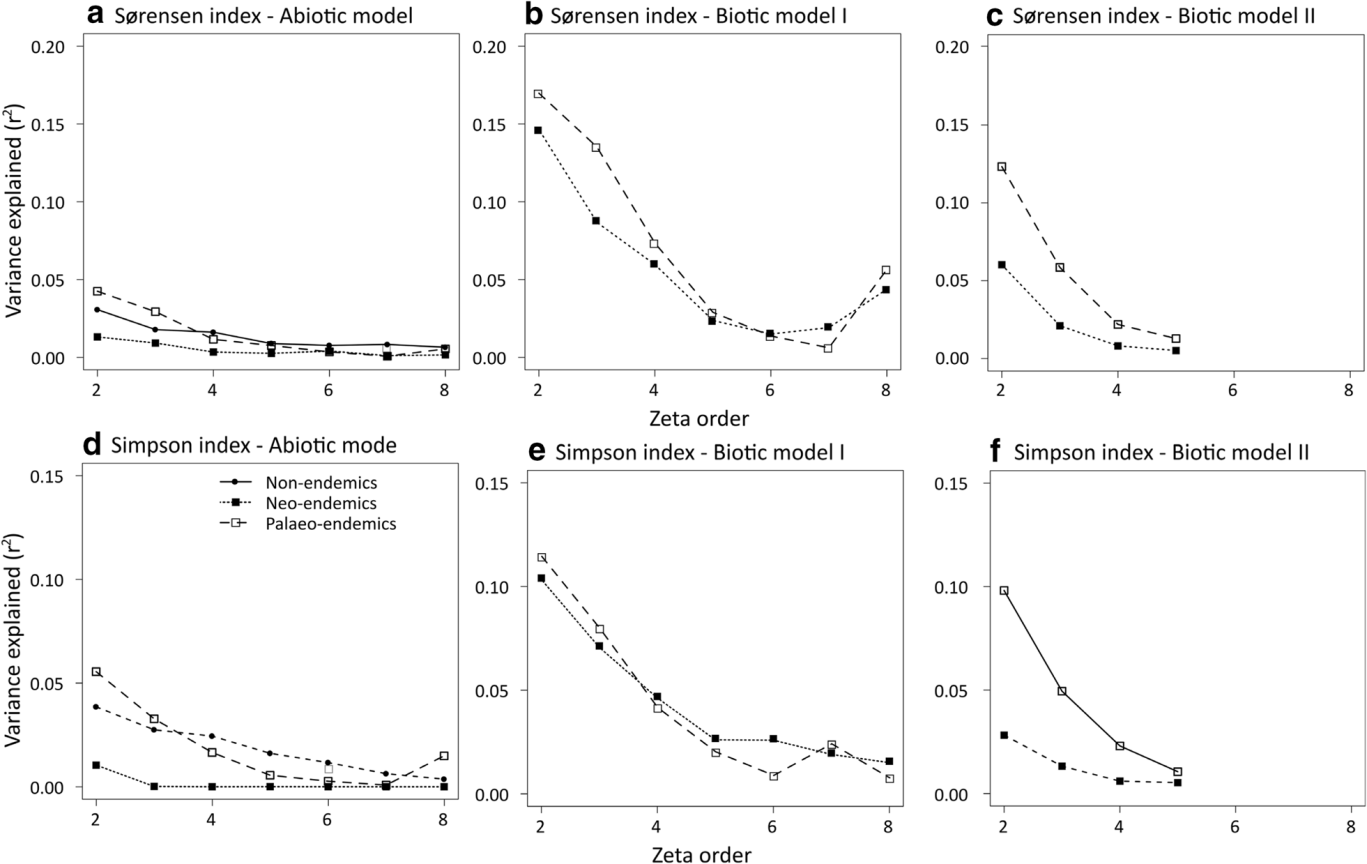
Variance explained by the abiotic Multi-Site Generalized Dissimilarity Model predicting Sørensen and Simpson zeta index differences (**a**, **d**) as function of environmental variables and distance for non-endemic, neo-endemic and palaeo-endemic vascular plants of Crete, Greece, and by the biotic I and II Multi-Site Generalized Dissimilarity Models for Sørensen (**b**, **e**) and Simpson (**c**, **f**) zeta index differences by adding the non-endemic zeta diversity, and palaeo-endemic or neo-endemic zeta diversity as predictors for neo-endemics and palaeo-endemics

The abiotic Multi-Site Generalized Dissimilarity Model (MS-GDM) including bioclimatic variables, human population density, and the percentage of human land uses explained a small part of the variation in Sørensen and Simpson zeta diversity indices, with the explained variance decreasing with zeta order (Fig. 2a). In the case of Sørensen index, variance explained was 0.031 for NON-E, 0.013 for NE and, and 0.042 for PE for zeta order equal to one. We observed similar low values for the Simpson index (Fig. 2b). Due to the low variance explained, the abiotic MS-GDMs provided little information about environmental and anthropogenic drivers of differences in species composition (Additional file 1: Figures S7–S12).

**Fig. 3:**
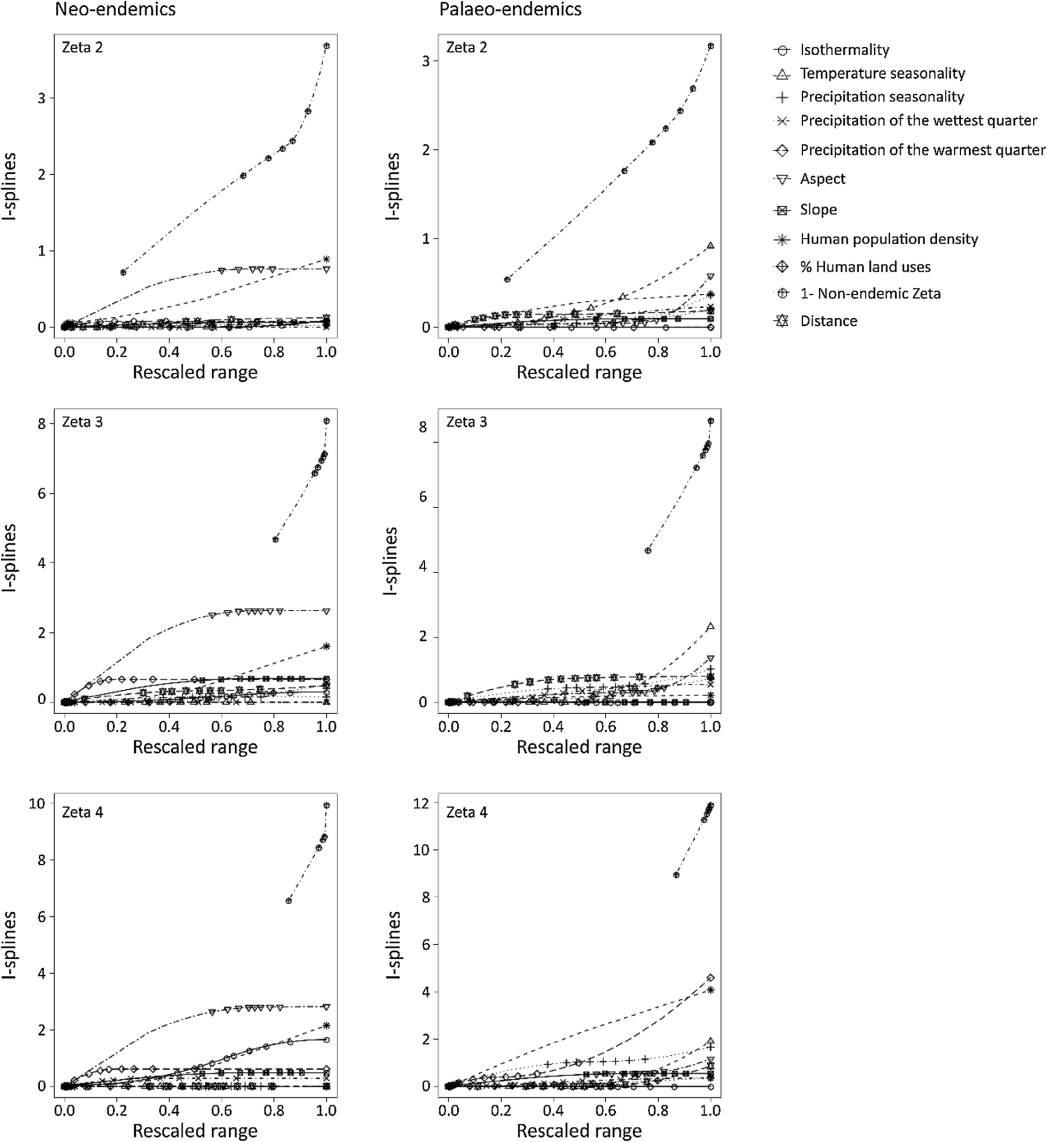
The effects of non-endemic zeta diversity, environmental variables and distance on differences in zeta diversity for different zeta orders estimated by Sørensen index for neo-endemic and palaeo-endemic vascular plants of Crete, Greece, as were estimated by multi-site generalized dissimilarity biotic model I. The predictors were transformed with I-splines

**Fig. 4:**
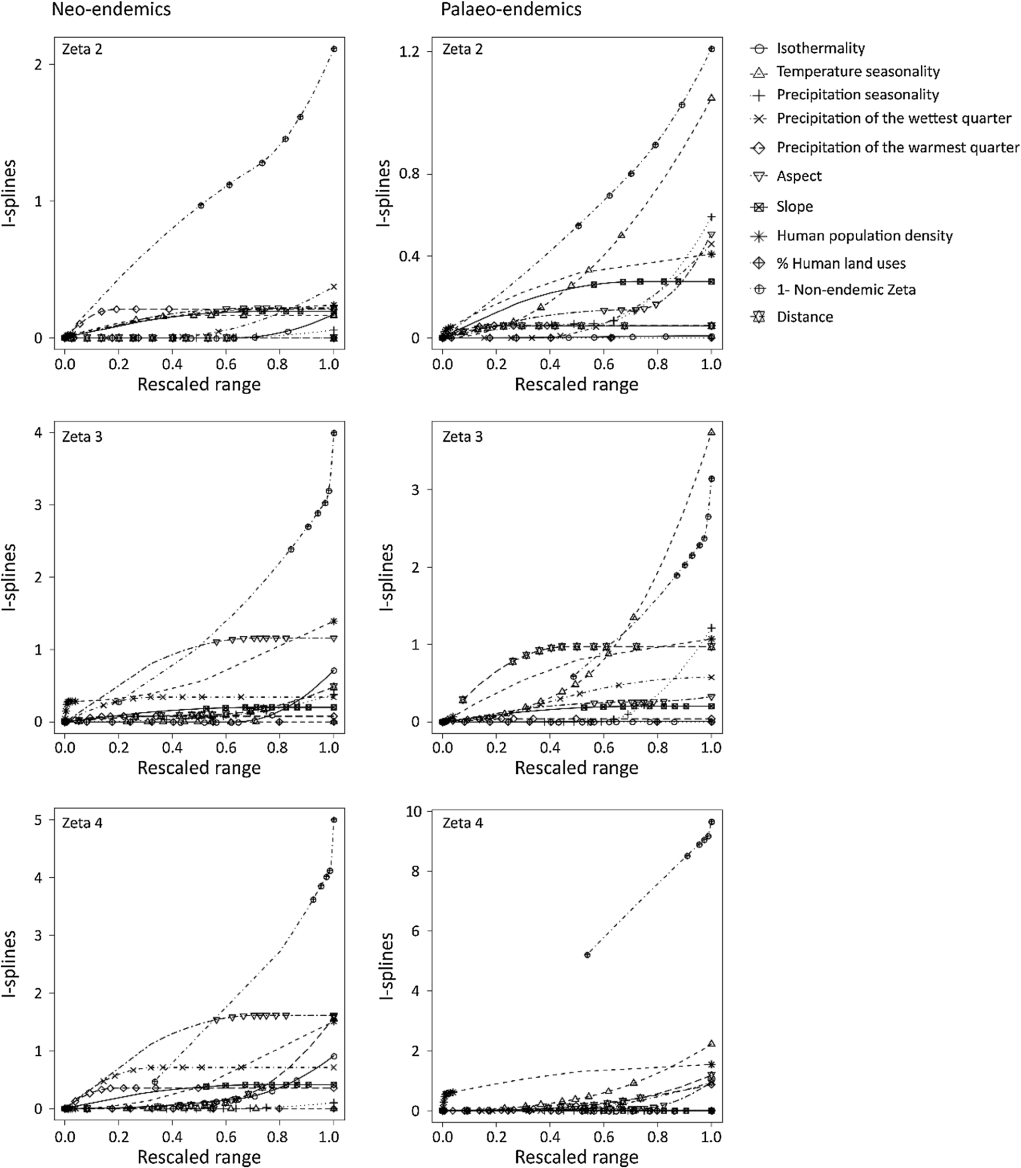
The effects of non-endemic zeta diversity, environmental variables and distance on differences in zeta diversity for different zeta orders estimated by Simpson index for neo-endemic and palaeo-endemic vascular plants of Crete, Greece, as were estimated by multi-site generalized dissimilarity biotic model I. The predictors were transformed with I-splines

The biotic model I MS-GDM i.e. the inclusion of zeta diversity of NON-E as predictor of differences in species turnover of NE and PE exhibited higher variance explained than the abiotic model at low zeta orders. The effect was slightly better for PE than NE, with variance explained for Sørensen index ranging between 0.001 and 0.15 (Simpson index: 0.008–0.11) for NE, and between 0.01 and 0.17 for PE (Simpson index: 0.003–0.12). The species composition of the NON-E had the highest effect on differences in NE and PE species composition. For NE Sørensen index, the biotic effect was followed in importance by topography (aspect, highest coefficient: first I-spline) and human population density (coefficient: third I-spline), while isothermality had an effect for zeta orders higher than 2 (highest coefficient: second I-spline) (Fig. 3). For PE Sørensen index, for zeta diversity of order 2 temperature seasonality (highest coefficient: third I-spline), topography (highest coefficient: third I-spline), and human population density (highest coefficient: second I-spline) contributed to differences in species composition (Fig. 3). With increasing order, we detected the effect of precipitation seasonality (highest coefficient: third I-spline) and precipitation of the warmest quarter (highest coefficient: second I-spline). Regarding Simpson index, apart from the prominent biotic effect, there was an effect of topography (aspect and slope, highest coefficient: third I-spline) and human population density (highest coefficient: third I-spline) for NE (Fig. 4). For PEs, species composition were also affected by temperature seasonality (highest coefficient: third I-spline), precipitation seasonality (highest coefficient: third I-spline), topography (aspect, highest coefficient: third and first I-spline for zeta order < 4 and zeta order = 4, respectively; slope, highest coefficient: first I-spline) and human population density (highest coefficient: second I-spline) (Fig. 4). The biotic model II for PE i.e. including NE zeta diversity as predictor of PE species turnover, exhibited higher variance explained than the abiotic model, but lower variance than the biotic model I. Biotic effect, distance and all environmental variables but temperature seasonality and human population density contributed to differences in PE Sørensen and Simpson index (Additional file 1: Figures S13, S14). However, for NE, the PE zeta diversity did not improve the performance of the MS-GDM (Additional file 1: Figures S13, S14).

## Discussion

### Elevational range and species distribution

Cretan flora consists of a similar number of palaeo-endemic and neo-endemic species. Our results demonstrated that NE exhibited higher mean elevation and minimum elevation of occurrence than PE, in concordance with Trigas et al. [35] suggesting that Cretan relict flora consists mostly of lowland species, while diversification at higher (middle) elevations gave rise to neo-endemic species. Not surprisingly, endemic species were more range-restricted than NON-E, while PE exhibited narrower, but not significantly, distributions than NE. There are many possible explanations for PE and NE narrow distributions. Palaeo-endemics are relicts of a past flora occurring in a fraction of their original distribution, and niche-based processes acting over a long time may have limited them in marginal environments for which they are well-adapted. Neo-endemics may have not fully expanded their distribution due to their relatively recent differentiation. Furthermore, range-restricted endemics may be low competitors with low dispersal ability [55], investing in local persistence rather than in high dispersal to survive [56, 57].

### α-diversity

There was a strong correlation between NE and PE α-diversity suggesting accumulation of endemics at similar sites across Crete i.e. centers of neo-endemism and palaeo-endemism may overlap, as it happens in the eastern part of the island. Regarding drivers of α-diversity, we found that climate, topography, and human effect were important for all categories, with the effect being stronger for PE. Local species richness of all species categories declined with human population density and showed a hump-shaped relationship with slope. Araújo [58] reported that human population density is positively correlated to plant species richness, but not related to species richness of narrow endemics in Europe. However, other studies found negative relationship between species richness and human population density [59, 60], in accordance with the observed pattern here. Kougioumoutzis and Tiniakou [61] found that human population density affected negatively the total number of endemic species of Cycladic islands in the Aegean area. According to Lavergne et al. [62], range-restricted endemic species in Mediterranean Basin are related to low human population density—often limited to inaccessible areas probably due to human pressure [45]—and slope. Furthermore, topographic relief is considered to promote endemic species richness [63], favoring neo-endemism through spatial divergence, but also palaeo-endemism in Mediterranean hotspots [57, 64]. Regarding climatic variables, we found that precipitation (precipitation seasonality for NE, and precipitation of wettest and warmest quarter for PE) shaped species richness patterns. The role of climate as determinant of species richness patterns has been extensively studied [65–68], especially in the face of climate change [69–71]. Molina-Venegas et al. [64] argue that palaeo-endemics tend to occur in wetter conditions than neo-endemics that are related to less benign environmental conditions.

### Species turnover

The species richness patterns and the geographical range size of different species categories were well reflected in their retention rate and the pattern of zeta diversity decline with zeta order. Specifically, PE with lowest species richness and narrower geographical range showed also a sharper decline of zeta diversity than NE and NON-E, and higher β-diversity as it was estimated by the N* index. Therefore, as zeta order increased, less PE than NE or NON-E species were shared between sites. This was more prominent in the low orders of zeta, reflecting the contribution of the rare species to the observed pattern. PE are ancient isolated species with small geographical range due to environmental change and habitat loss. Perhaps, the environmental change has rendered their traits less adaptive to the present-day environmental conditions [72], with PE being often habitat specialists restricted to specific environmental conditions. These species may have persisted in refugia [73] e.g. ecosystems at high elevations [35]. Mountainous topography favors high endemism [73] e.g. cliffs in the Mediterranean are considered refugia for plant species with unique species composition [74]. On the other hand, NE exhibited significantly lower species richness and narrower geographical range than NON-E. A possible explanation could be that NE may have not dispersed very far reaching their full potential in terms of geographical range [75] or they are of lower competitive ability [55]. Finally, the zeta diversity decline for rare species was described by both power-law and negative exponential model, indicating that deterministic processes and stochasticity drive species turnover of rare species. The deterministic processes were stronger for NON-E and NE (greater power-law coefficient), while stochasticity and deterministic processes contributed similarly to rare PE turnover. The latter finding may be interpreted by the role of environmental stochasticity in shaping PE turnover (see below). Contrarily, the turnover pattern of widespread species was driven by stochasticity, as also noted by Latombe et al. [27] who found that species turnover of rare vascular plants with different residence time is driven by both deterministic and stochastic processes, whereas stochasticity prevails in the case of widespread species.

The environmental and anthropogenic variables were strong determinants of species richness i.e. for zeta diversity of order 1 (ζ_1_), but the variance explained of the abiotic MS-GDM predicting species turnover was low across zeta orders, with slightly better performance for PE. The declining explanatory power of predictors from ζ_1_ (species richness) to higher orders indicates that different drivers shape species richness and species turnover of vascular plants in Crete. Perhaps, environmental variables not considered here are better predictors of species turnover e.g. soil properties are considered strong drivers of β-diversity patterns [76], especially for the heterogeneous Mediterranean ecosystems [77].

Biotic effects in the Biotic model I (zeta diversity of NON-E as predictor of NE and PE turnover) increased the variance explained by the MS-GDM, especially for rare species. The variance explained increased more in the case of the richness-dependent Sørensen index. This was also indicated from the significant correlation of species richness (ζ_1_) of NON-E with both NE and PE species richness. The incorporation of biotic effects has shown that improve significantly the predictive power of species distribution models of plants across spatial scales [78–80], and our results suggest that this may apply to turnover also. These biotic effects can be negative plant–plant interactions e.g. competition affecting species distribution and composition [81–83] or positive e.g. facilitation of expansion of species distribution toward higher elevations [84]. Although increased values of C-score provided evidence for disaggregation, the impact of biotic effects on turnover remains an open question and further research on the co-occurrence patterns is required. However, the predictive power of MS-GDM diminished for widespread species, suggesting that turnover of widespread NE and PE is independent of the species composition of NON-E. The environmental and anthropogenic variables exerting significant effect on species richness appeared to have a role on the richness-dependent turnover i.e. topography and human population for NE, and additionally, temperature seasonality and precipitation related variables for PE. Similar patterns were observed in the case of Simpson index. Human population density was more important when it was intermediate for NE or high for PE (i.e. urbanization effect). Furthermore, seasonality was significant for PE when it was high. The link between turnover and seasonality in Mediterranean landscapes has been highlighted for vertebrates by Martin and Ferrer [85], and perhaps this applies also to plants. It is highly probable that many palaeo-endemics may have emerged before the establishment of typical Mediterranean climate [34] and their distribution is now restricted at high elevations where the climate resembles the pre-Mediterranean one with low (or no) seasonality. On the other hand, biotic effects in model II (zeta diversity of NE as predictor of PE and vice versa) increased the variance explained by the MS-GDM only for PE, and once again especially for rare species. It seems that only species distribution of rare PE relates to the species distribution of NE. A possible explanation could be that hotspots of NE are in areas where the habitats are suitable also for PE. A potential limitation of our study is the classification of endemic species into palaeo-endemics and neo-endemics according to published studies and the systematic isolation criterion. Recent studies have attempted to classify neo-endemics and palaeo-endemics using time-calibrated phylogenetic trees. This approach might lead to differences in the observed patterns, thus future research incorporating phylogenetic information could shed more light on the diversity patterns of endemism.

## Conclusions

The spatial patterns of neo-endemic and palaeo-endemic richness imply that hotspots of neo-endemism coincide with palaeo-endemism hotspots, perhaps due to the effect of climate, topography, and anthropogenic variables on α-diversity. More interesting were the species turnover patterns which highlighted the contribution of rare species to the observed patterns. The profile of zeta diversity decline with zeta order revealed that palaeo-endemics showed a sharper decline, followed by neo-endemics and then by non-endemics. A possible explanation could be that palaeo-endemics, as relict species less adapted to current environmental conditions and often habitat specialists, exhibited narrow distributions, and thus the number of shared species across sites decreased sharply. The narrow distribution of neo-endemics is restricted due to recent differentiation. Despite the significant effect of environmental and anthropogenic variables on α-diversity, their effect on zeta diversity was weak implying that different drivers affect the spatial patterns of species richness and species turnover of vascular plants in Crete. Finally, our analysis emphasized the role of biotic effects in driving turnover patterns.

## Supplementary information

**Additional file 1. Figure S1** (i) the generated timed phylogeny analysis of the vascular plants of Crete used in our study, **Figures S2–S6** (ii) the species richness patterns and their drivers of non-endemic, neo-endemic and palaeo-endemic species, and **Figures S7–S14** (iii) the I-splines of abiotic (predictors: bioclimatic variables, human population density and the percentage of human land uses) and biotic II (predictors: predictors of abiotic model and neo-endemic zeta diversity as predictor of palaeo-endemic zeta diversity and vice versa) Multi-Site Generalized Dissimilarity Models showing the contribution of different predictors to explaining zeta diversity of different species categories

## Data Availability

The biodiversity dataset used/analyzed during the current study is published in Turland et al. [42], the climate and the land use data were derived from WorldClim dataset [40] and CORINE Land Cover 2000 database (Copernicus land monitoring services 2018) respectively. All sources are publicly available.
