# Supplementary material for: Patterns and drivers of species richness and turnover of neo-endemic and palaeo-endemic vascular plants in a Mediterranean hotspot: the case of Crete, Greece
Source: J Biol Res (Thessalon). 2019 Nov 5;26:12. doi: 10.1186/s40709-019-0106-x (PMC6833306; doi:10.1186/s40709-019-0106-x)
Supplement: Supplementary file 1 — Additional file 1. Figure S1 (i) the generated timed phylogeny analysis of the vascular plants of Crete used in our study, Figures S2–S6 (ii) the species richness patterns and their drivers of non-endemic, neo-endemic and palaeo-endemic species, and Figures S7–S14 (iii) the I-splines of abiotic (predictors: bioclimatic variables, human population density and the percentage of human land uses) and biotic II (predictors: predictors of abiotic model and neo-endemic zeta diversity as predictor of palaeo-endemic zeta diversity and vice versa) Multi-Site Generalized Dissimilarity Models showing the contribution of different predictors to explaining zeta diversity of different species categories [file 40709_2019_106_MOESM1_ESM.docx]

Additional Material

**Patterns and drivers of species richness and turnover of neo-endemic and palaeo-endemic vascular plants in a Mediterranean hotspot: the case of Crete, Greece**

Authors: Maria Lazarina^1^*, Athanasios S. Kallimanis^1^, Panayotis Dimopoulos^2^, Maria Psaralexi^1^, and Stefanos P. Sgardelis^1^

Addresses:

^1^Department of Ecology, School of Biology, Aristotle University, 54124 Thessaloniki, Greece. Maria Lazarina: mlazarin@bio.auth.gr, Athanasios S. Kallimanis: kalliman@bio.auth.gr, Maria Psaralexi: mpsarale@bio.auth.gr, Stefanos P. Sgardelis: sgardeli@bio.auth.gr

^2^Department of Biology, Laboratory of Botany, University of Patras, GR‐26504 Rio, Patras, Greece. Panayotis Dimopoulos: pdimopoulos@upatras.gr

*Correspondence to: Maria Lazarina, Department of Ecology, School of Biology, Aristotle University, 54124, Thessaloniki, Greece. mlazarin@bio.auth.gr

*Short description of the analysis performed to generate timed phylogeny for the species included in our analysis*

We explored which of the species of our analysis are included in the three most comprehensive and commonly used databases providing time-calibrated phylogenies, i.e. TimeTree (1) cited in 274 publications, PhytoPhylo (2) cited in 109 papers, and GBOTB (3) cited 38 times, standardizing the botanical nomenclature according to the Plant List using the R package Taxonstand (4). However, a major issue in plant megalphylogenies is the incompleteness with high proportion of unresolved topologies when the phylogenetic tree involves many under-sampled taxa (5, 6). The phylogeny provided by TIMETREE website included 34 species and no subspecies out of the 165 taxa in our analysis and had 4 polytomies (Fig.S1), while providing no information for the remaining species or subspecies. The data provided by Smith and Brown (3) were accessed via the V.PhyloMaker R package (7). V.PhyloMaker matched 59 species out of the 157 input species, binding the remaining arbitrarily or systematically to the tree. This method leads to inaccurate age estimations (either over- or under-estimations), as species not present in the backbone phylogeny and lacking sequence data, are assigned at equal distances with all the other species of the same genus. Thus, these species are estimated to have the same divergence age as other species of the genus, which is highly unlikely (8). This approach resulted in highly unresolved phylogenies at species level with improbable age estimations, especially in genera with many species, e.g. Allium, Alyssum, Campanula, Centaurea, and Hypericum. Furthermore, none of the subspecies (38 subspecies) which we defined priori neoendemics was included, as the package does not allow for information below species level and combines them with their parental species. As a result, their divergence is overestimated as species are by definition older than the subspecies of our dataset. PhytoPhylo phylogeny, accessed via the S.PhyloMaker function, contained only 17 species from our species list and we did not try to build a timed tree since it would also have many polytomies.


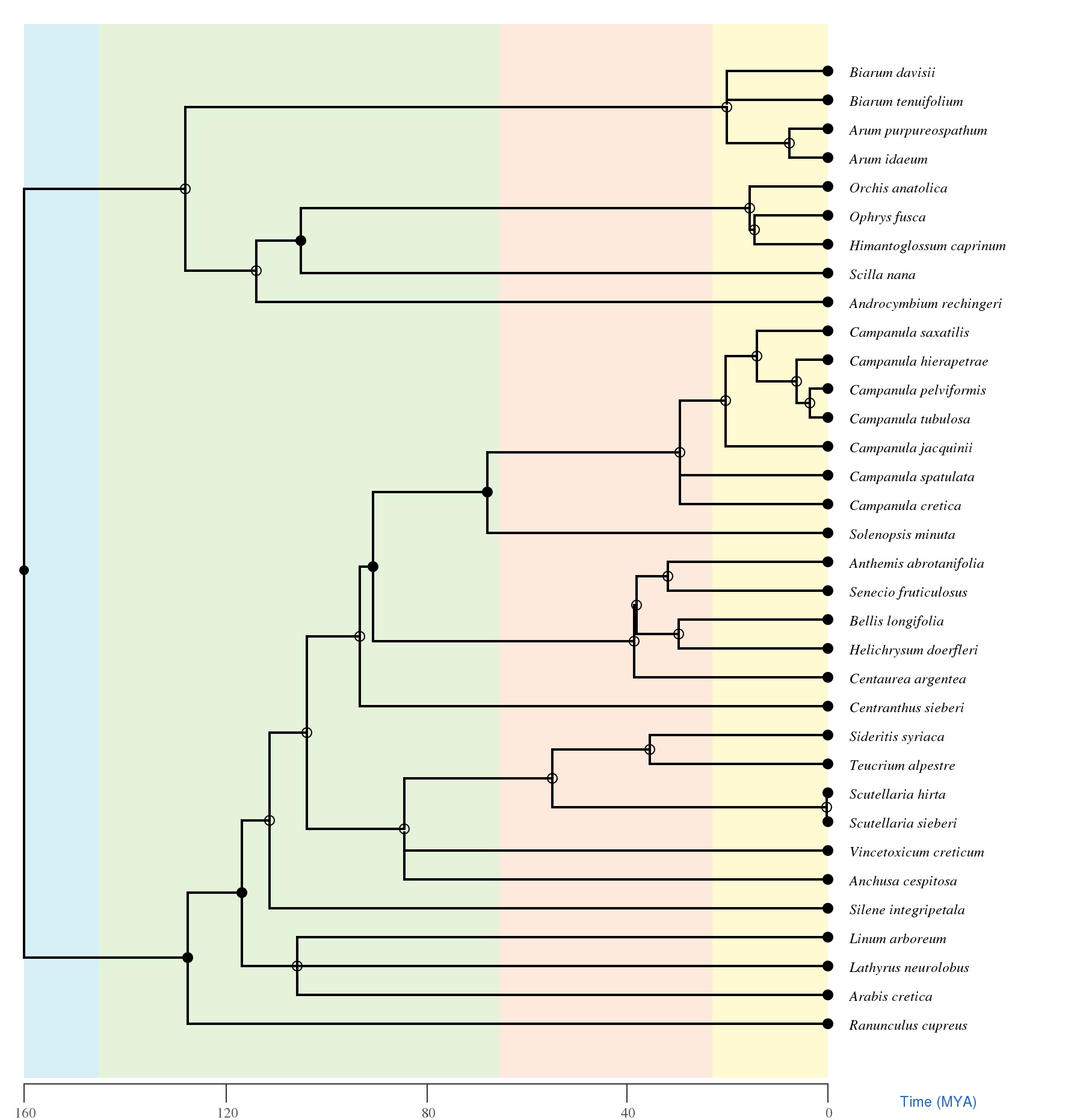


Figure S1. The timed phylogeny generated by TimeTree website. The background colours represent geologic periods. Full circles indicate nodes that map directly to the NCBI Taxonomy and the open circles indicate nodes that were created during the polytomy resolution process which is described in Hedges, Marin (9).


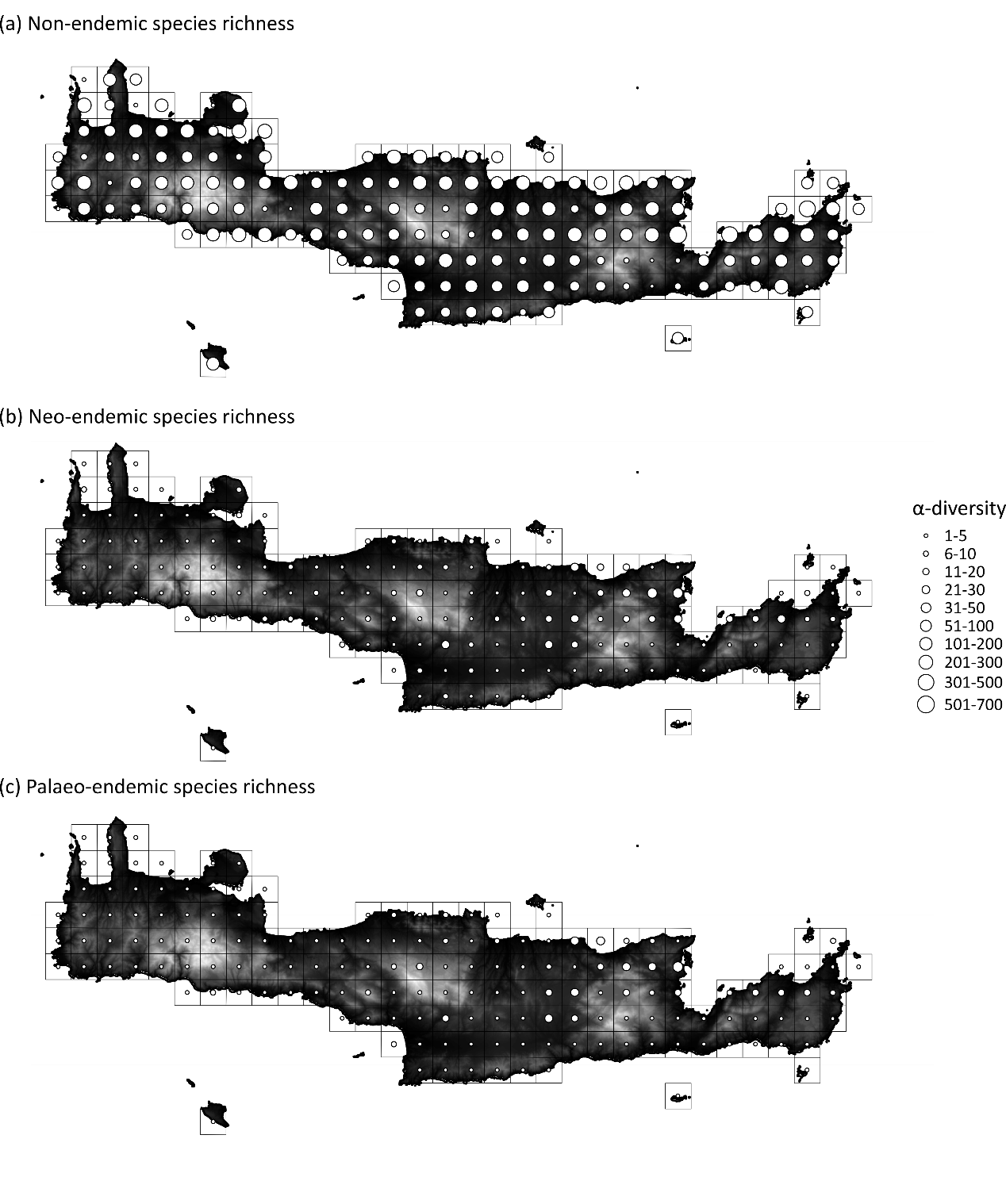
Figure S2. α-diversity i.e. species richness at site level of non-endemic, neo-endemic and palaeoendemic vascular plants of Crete, along with the variation of elevation.


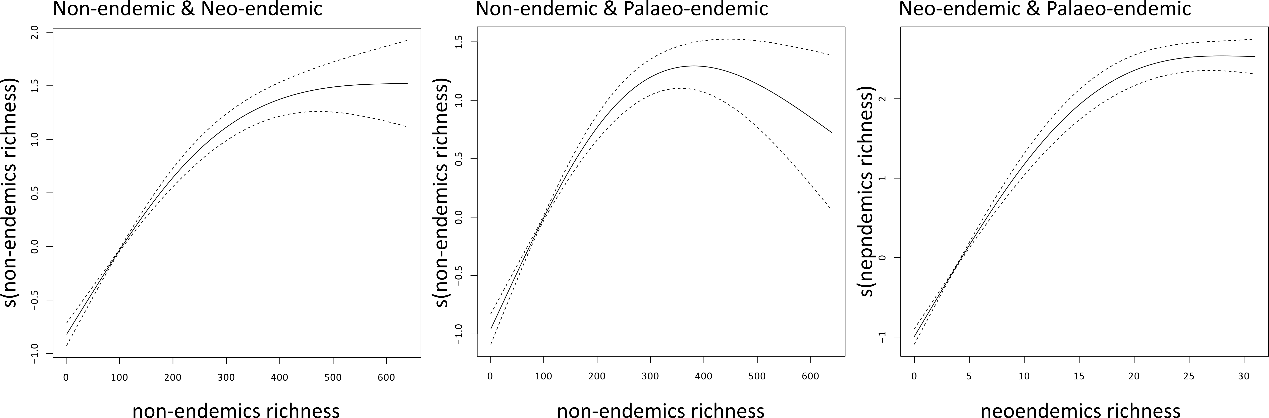


Figure S3. The relationship between non-endemic, neoendemic and paleoendemic richness pairwisely modelled by Generalized Additive Models for vascular plants of Crete, Greece.


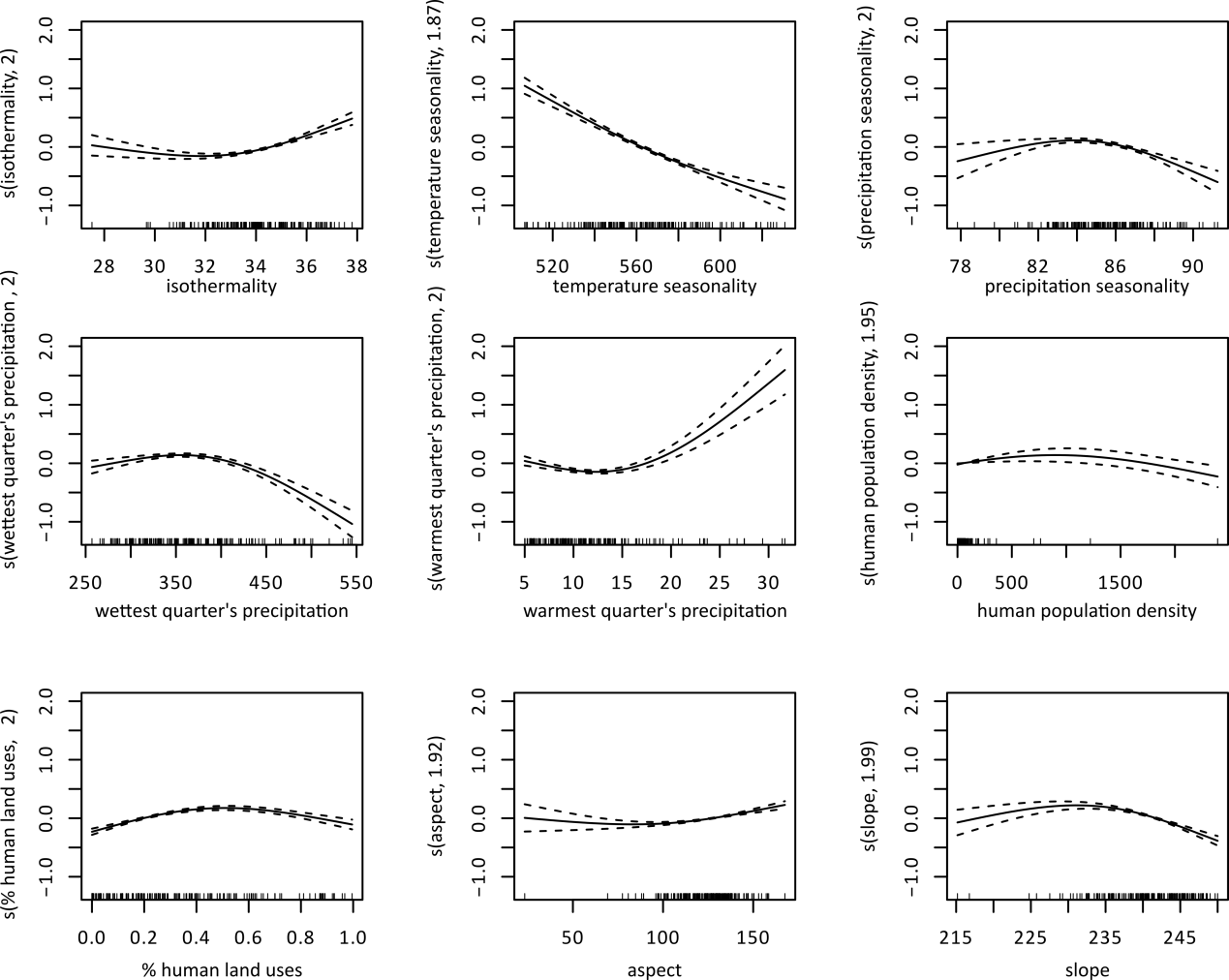


Figure S4. Effect plots showing the results of formulated Generalized Additive Models predicting non-endemic richness of vascular plants of Crete, Greece, as function of bioclimatic, topographical and human effect variables (smooth terms) after accounting for spatial autocorrelation. Only significant effects are presented.


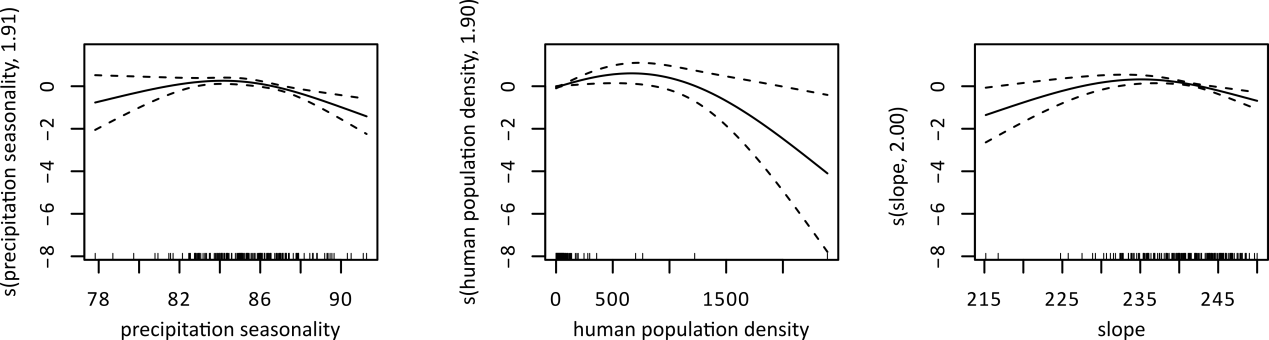


Figure S5. Effect plots showing the results of formulated Generalized Additive Models predicting neo-endemic richness of vascular plants of Crete, Greece, as function of bioclimatic, topographical and human effect variables (smooth terms) after accounting for spatial autocorrelation. Only significant effects are presented.


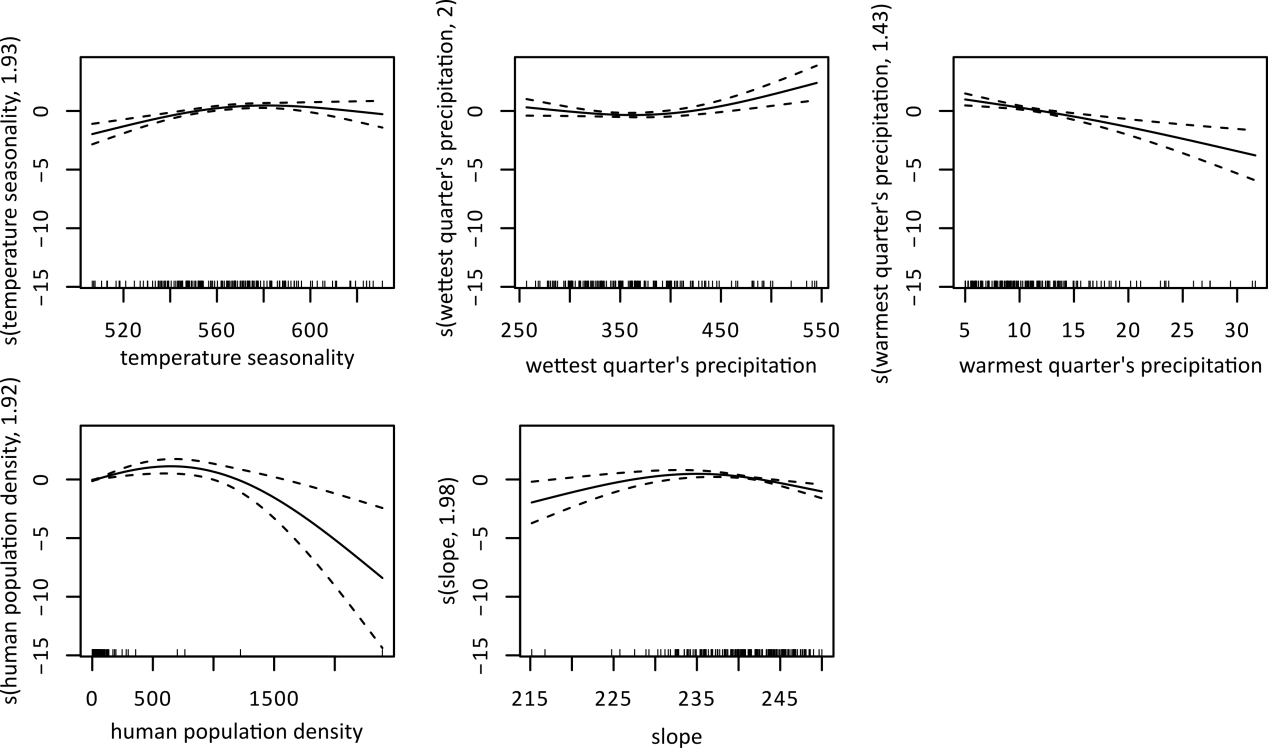


Figure S6. Effect plots showing the results of formulated Generalized Additive Models predicting palaeo-endemic richness of vascular plants of Crete, Greece, as function of bioclimatic, topographical and human effect variables (smooth terms) after accounting for spatial autocorrelation. Only significant effects are presented.


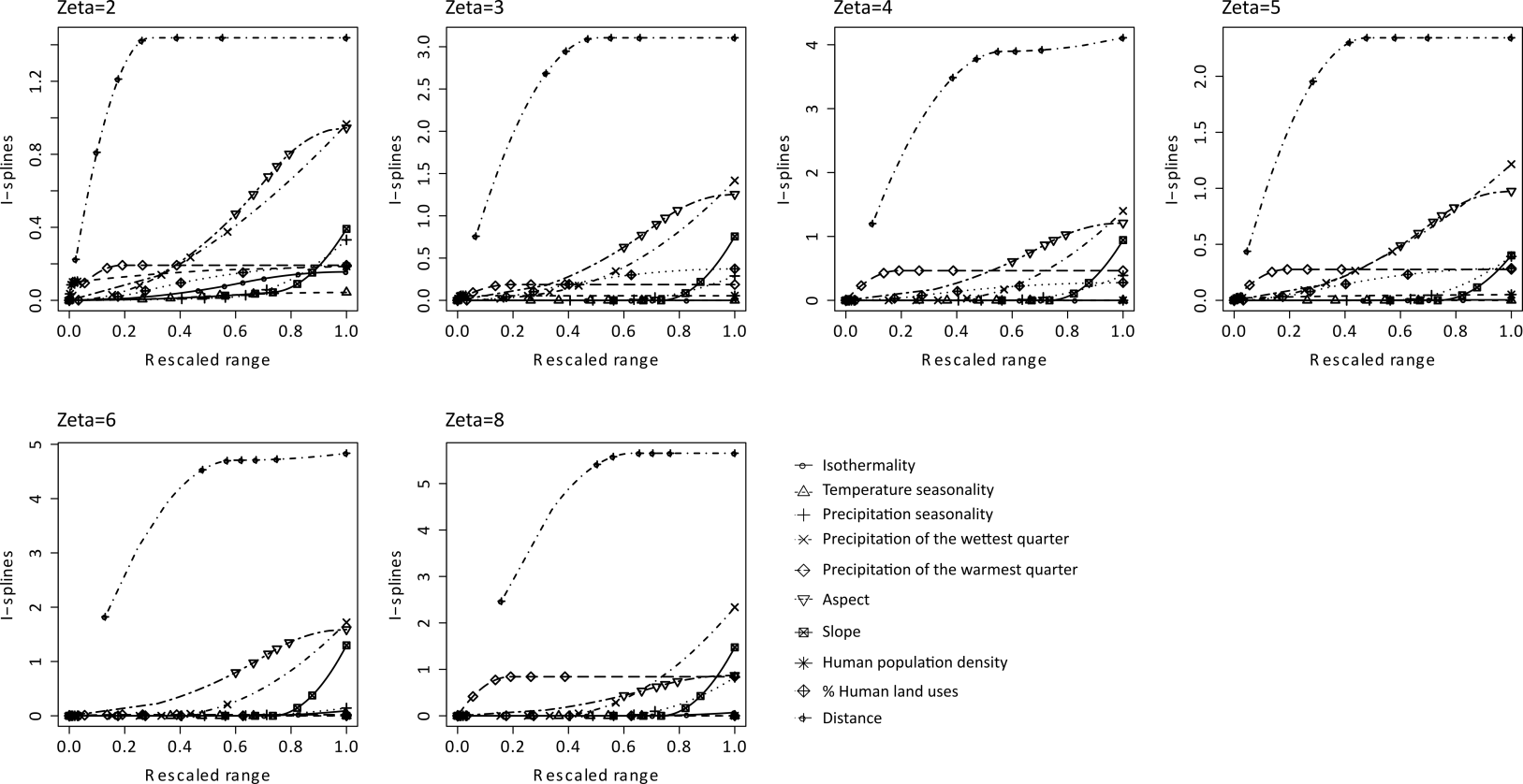


Figure S7. The effects of environmental variables and distance on differences in zeta diversity for different zeta orders estimated by Sorensen index for non-endemics vascular plants of Crete, Greece, as were estimated by multi-site generalized dissimilarity abiotic model. The predictors were transformed with I-splines.


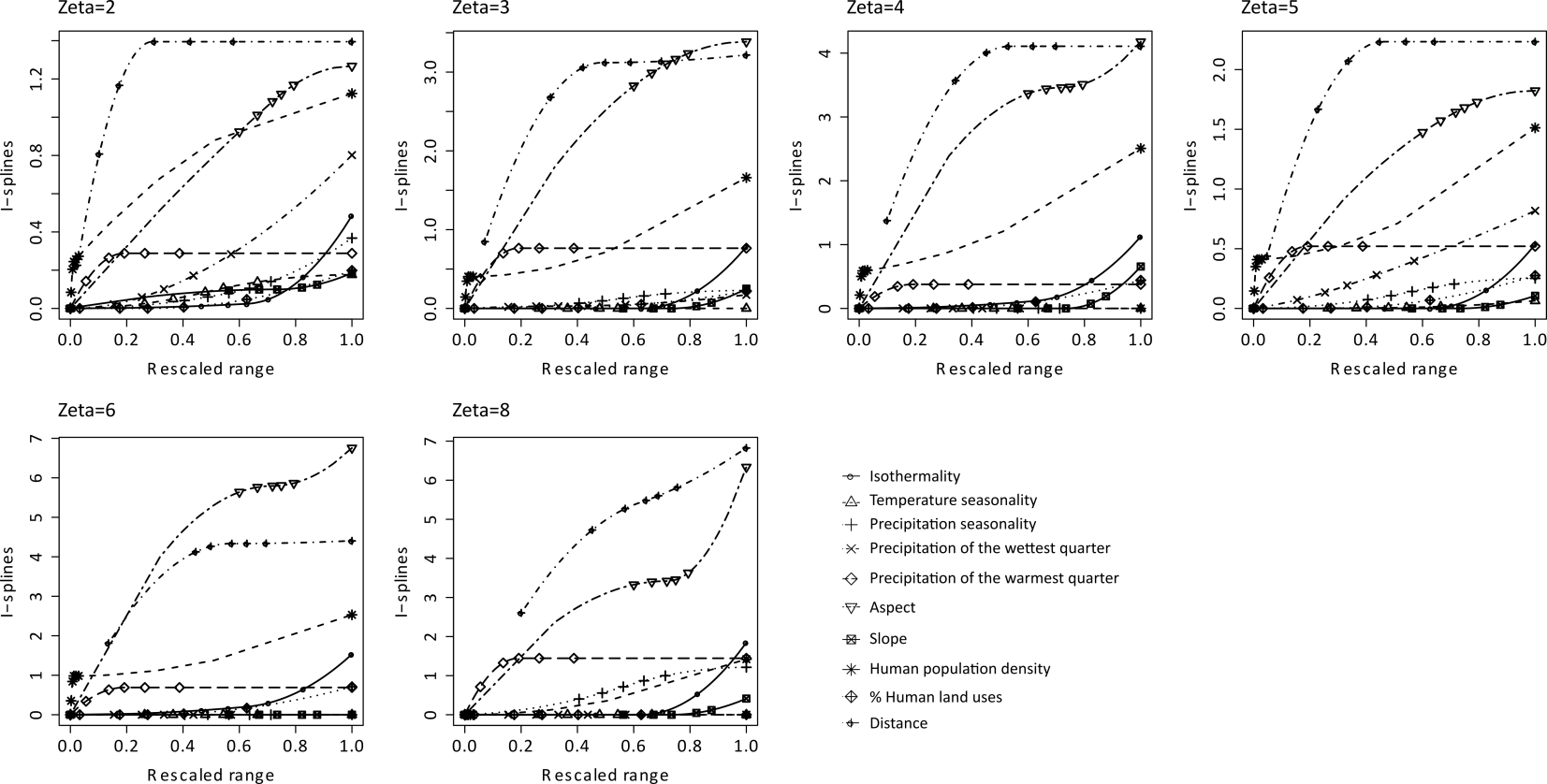


Figure S8. The effects of environmental variables and distance on differences in zeta diversity for different zeta orders estimated by Sorensen index for neo-endemic vascular plants of Crete, Greece, as were estimated by multi-site generalized dissimilarity abiotic model. The predictors were transformed with I-splines.


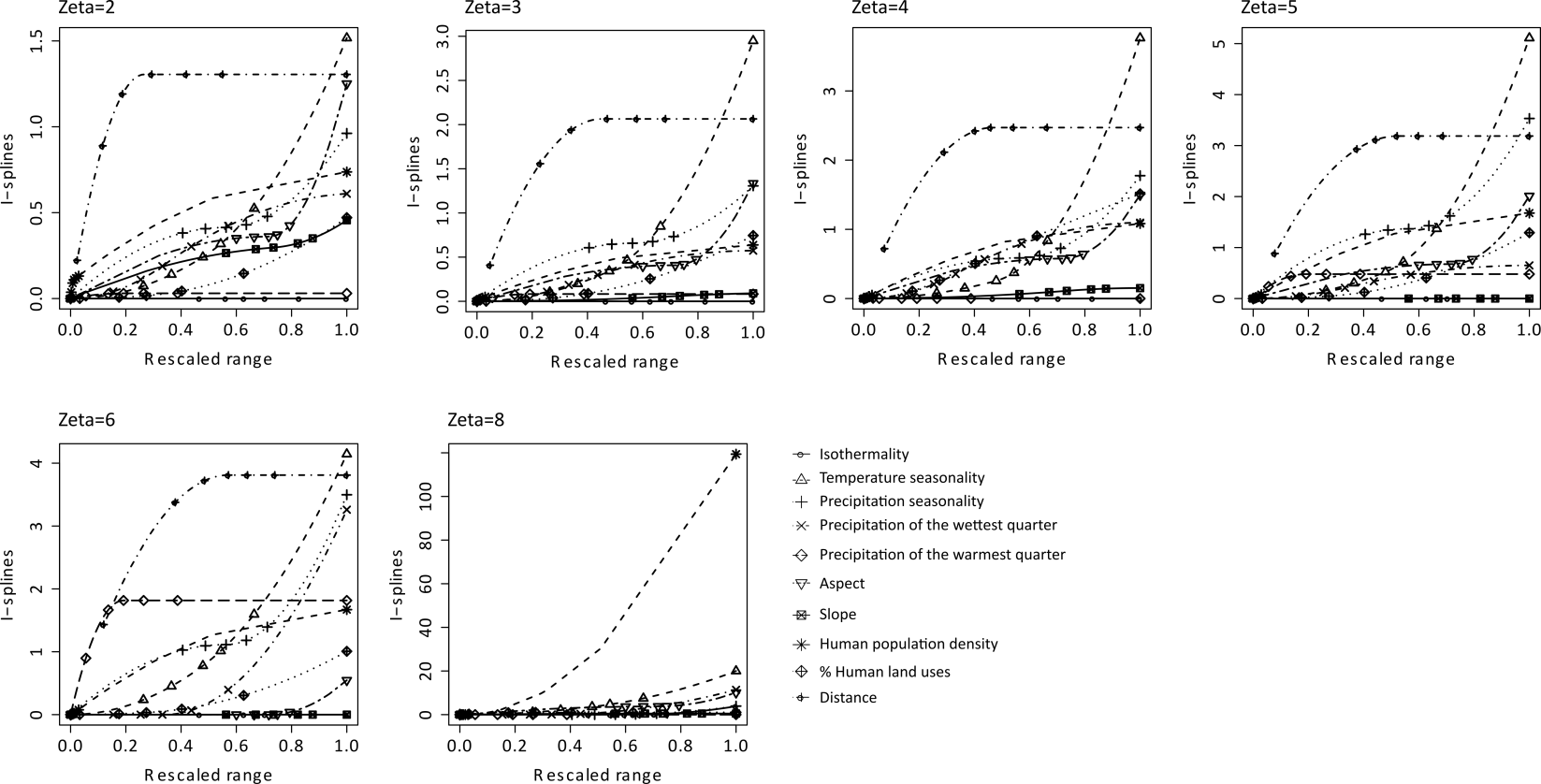


Figure S9. The effects of environmental variables and distance on differences in zeta diversity for different zeta orders estimated by Sorensen index for palaeo-endemic vascular plants of Crete, Greece, as were estimated by multi-site generalized dissimilarity abiotic model. The predictors were transformed with I-splines.


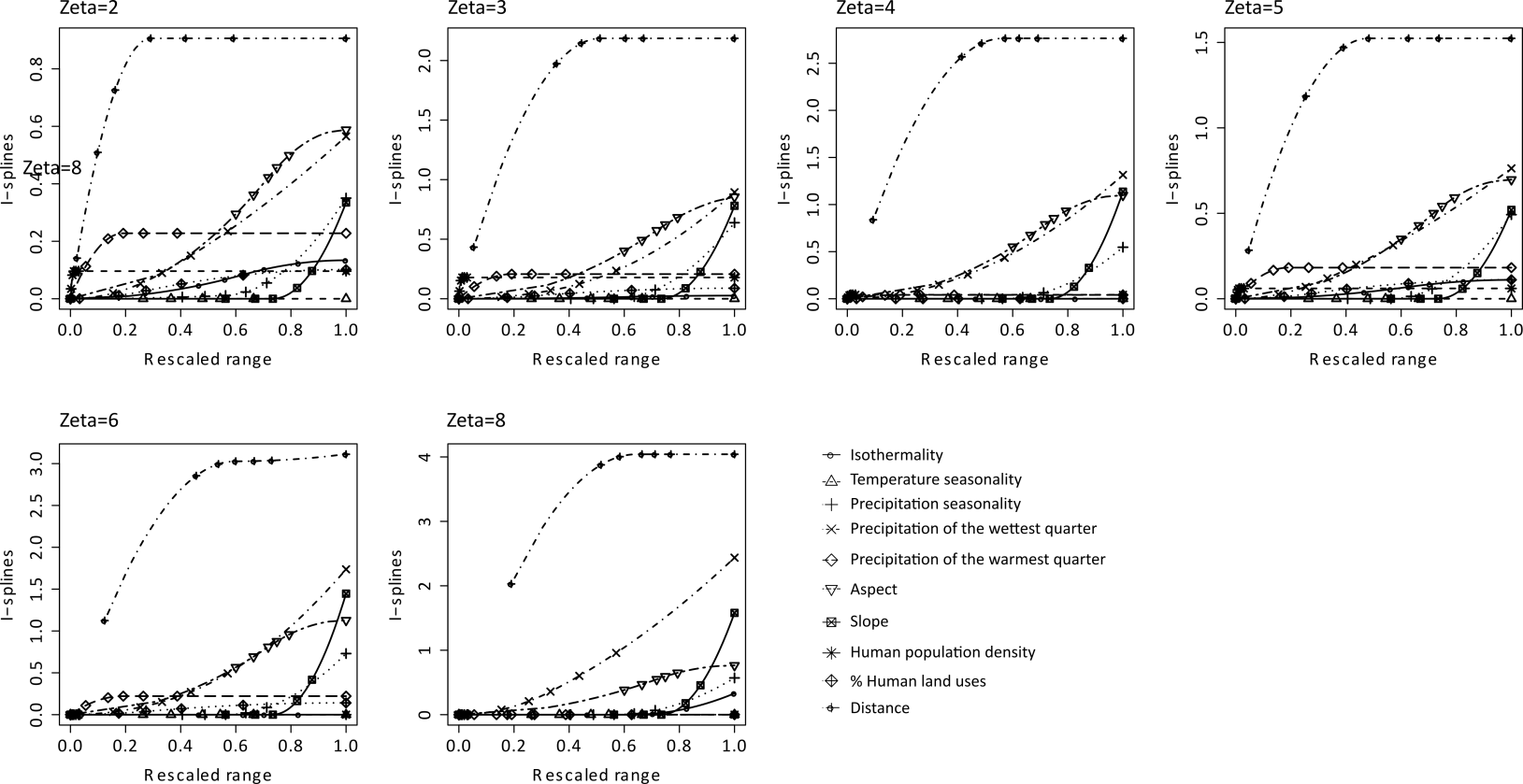


Figure S10. The effects of environmental variables and distance on differences in zeta diversity for different zeta orders estimated by Simpson index for non-endemic vascular plants of Crete, Greece, as were estimated by multi-site generalized dissimilarity abiotic model. The predictors were transformed with I-splines.


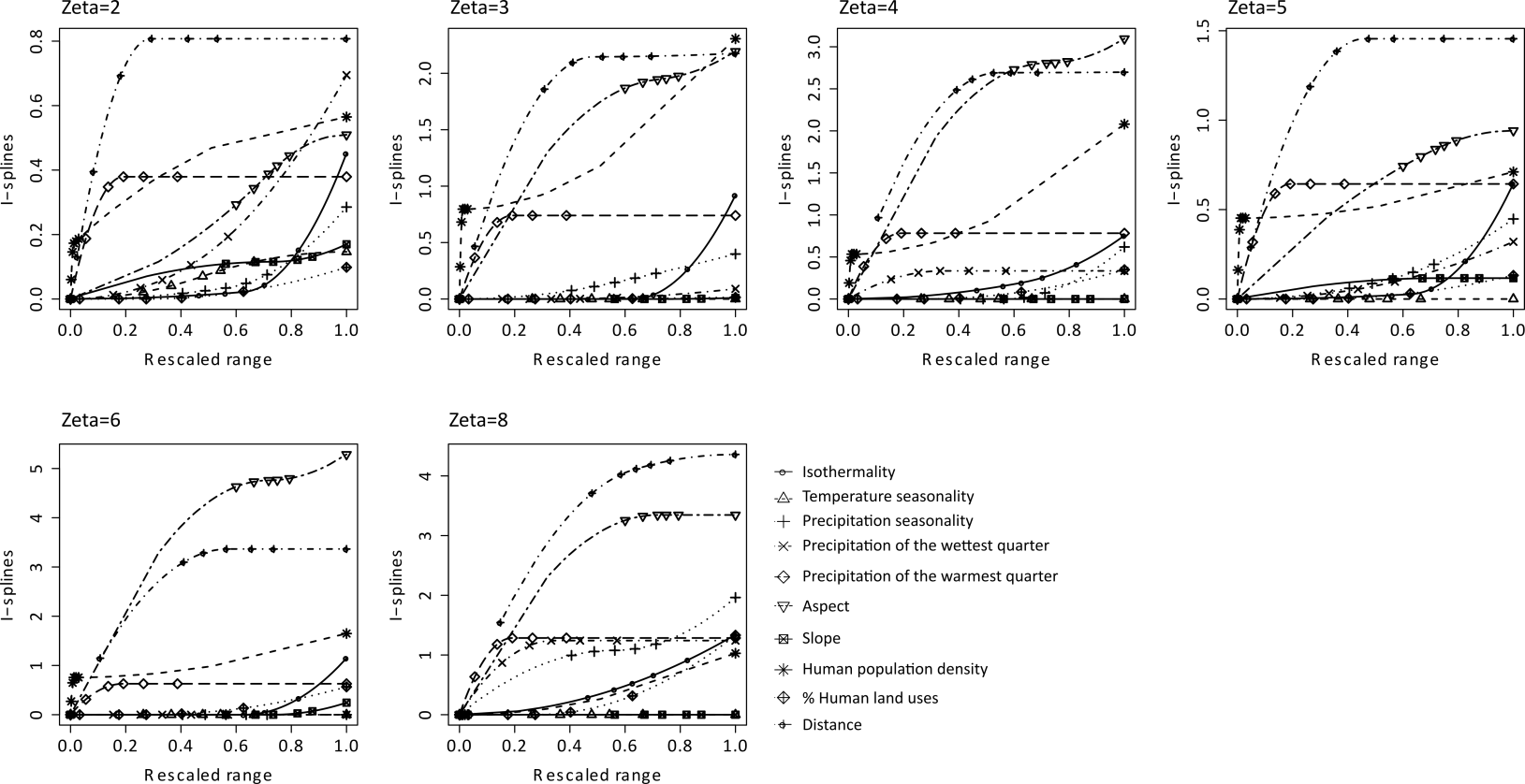


Figure S11. The effects of environmental variables and distance on differences in zeta diversity for different zeta orders estimated by Simpson index for neo-endemic vascular plants of Crete, Greece, as were estimated by multi-site generalized dissimilarity abiotic model. The predictors were transformed with I-splines.


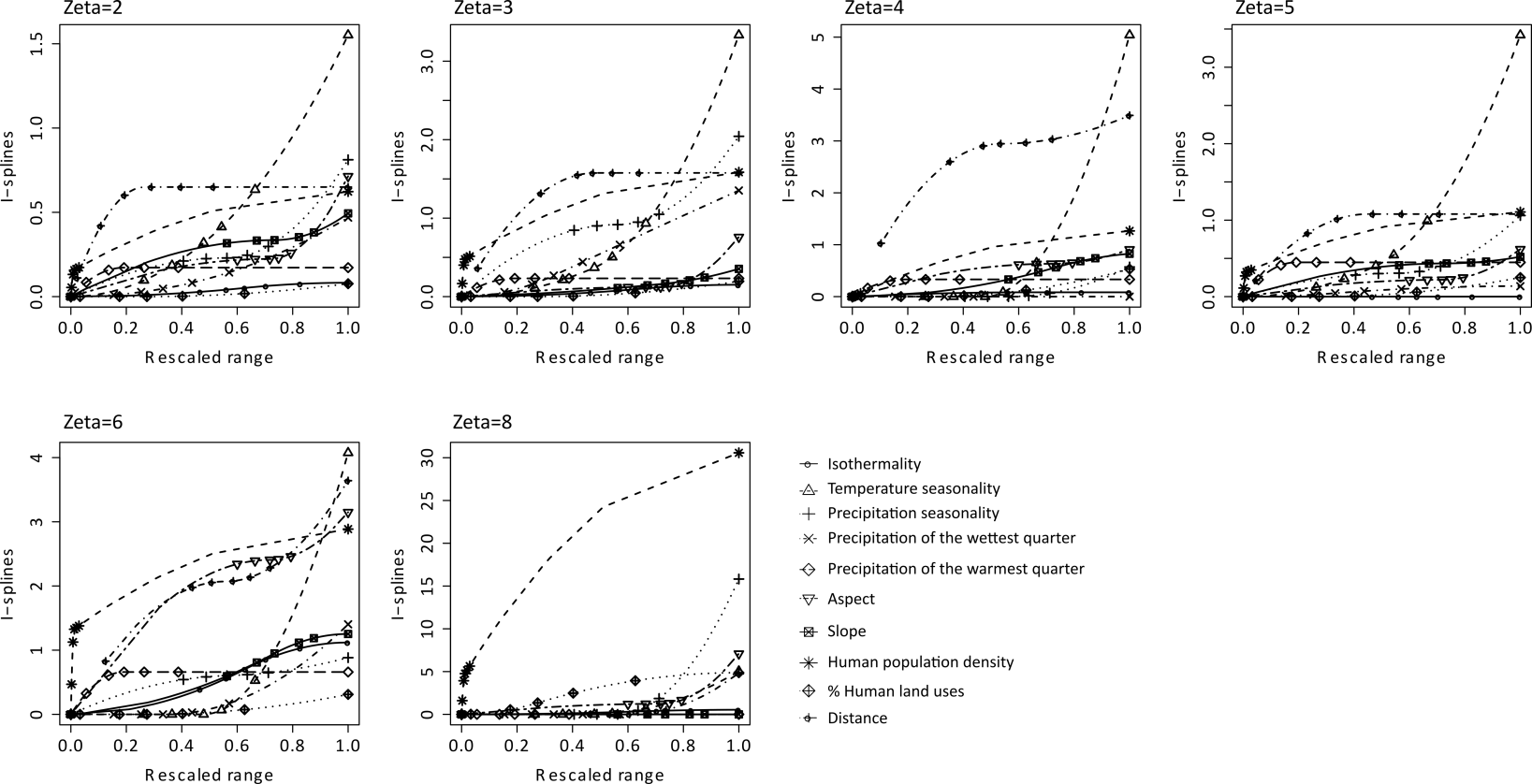


Figure S12. The effects of environmental variables and distance on differences in zeta diversity for different zeta orders estimated by Simpson index for palaeo-endemic vascular plants of Crete, Greece, as were estimated by multi-site generalized dissimilarity abiotic model. The predictors were transformed with I-splines.


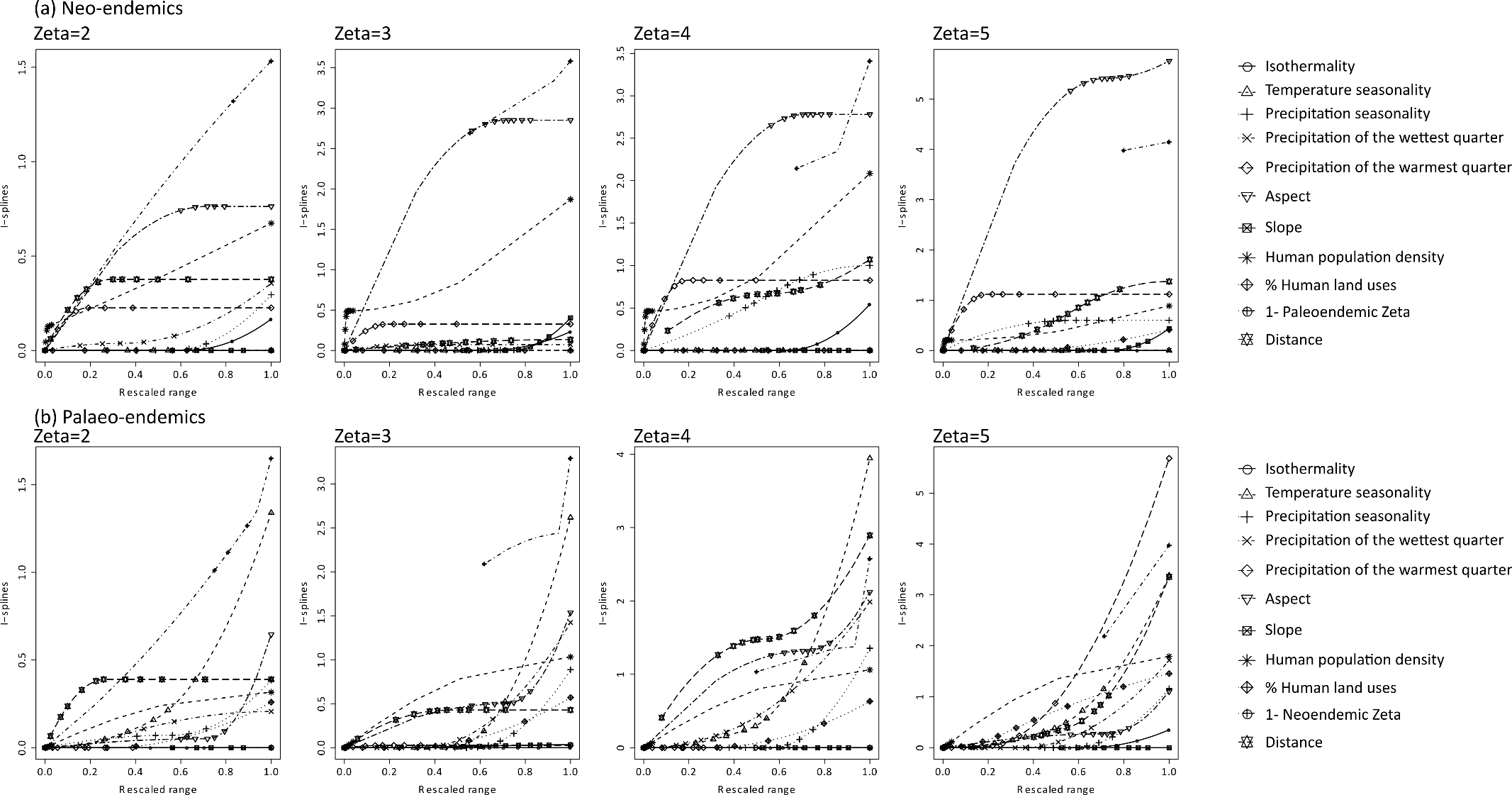


Figure S13. The effects of environmental variables, distance and paleoendemic zeta diversity for neoendemic, and neoendemic zeta diversity for paleoendemic on differences in zeta diversity estimated by Sorensen index for neoendemic (a) and paleoendemic (b) vascular plants of Crete, Greece, for different zeta orders as were estimated by multi-site generalized dissimilarity biotic model II. The predictors were transformed with I-splines.


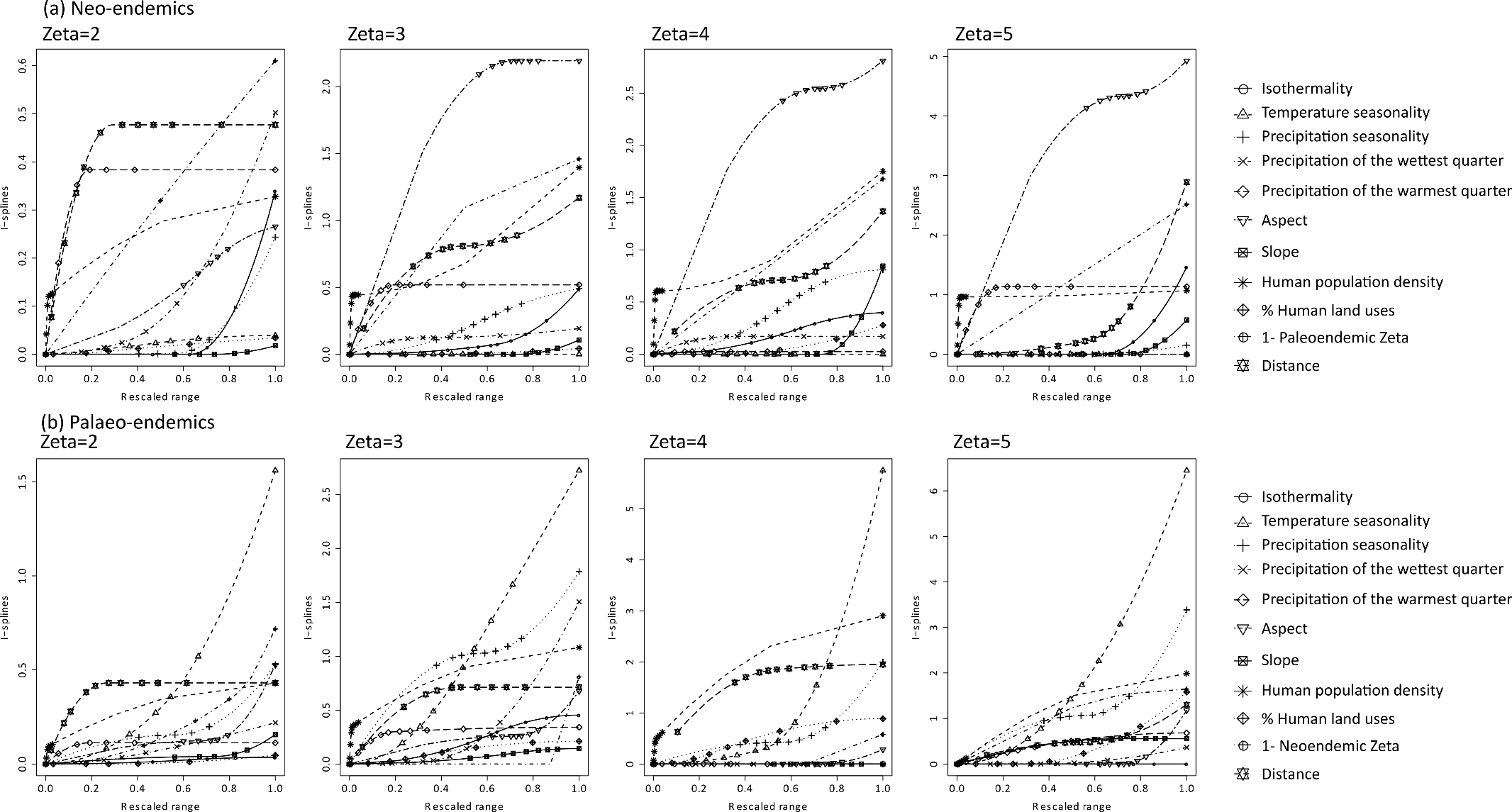
 Figure S14. The effects of environmental variables, distance and paleoendemic zeta diversity for neoendemic and neoendemic zeta diversity for paleoendemic on differences in zeta diversity estimated by Simpson index for neoendemic (a) and paleoendemic (b) vascular plants of Crete, Greece, for different zeta orders as were estimated by multi-site generalized dissimilarity biotic model II. The predictors were transformed with I-splines.

**References**

1. Kumar S, Stecher G, Suleski M, Hedges SB. TimeTree: a resource for timelines, timetrees, and divergence times. Molecular biology and evolution. 2017;34(7):1812-9.

2. Qian H, Jin Y. An updated megaphylogeny of plants, a tool for generating plant phylogenies and an analysis of phylogenetic community structure. Journal of Plant Ecology. 2016;9(2):233-9.

3. Smith SA, Brown JW. Constructing a broadly inclusive seed plant phylogeny. American Journal of Botany. 2018;105(3):302-14.

4. Cayuela L, Granzow‐de la Cerda Í, Albuquerque FS, Golicher DJ. Taxonstand: An R package for species names standardisation in vegetation databases. Methods in Ecology and Evolution. 2012;3(6):1078-83.

5. Li D, Trotta L, Marx HE, Allen JM, Sun M, Soltis DE, et al. For common community phylogenetic analyses, go ahead and use synthesis phylogenies. Ecology. 2019:e02788.

6. Beaulieu JM, Ree RH, Cavender-Bares J, Weiblen GD, Donoghue MJ. Synthesizing phylogenetic knowledge for ecological research. Ecology. 2012;93(sp8):S4-S13.

7. Jin Y, Qian H. V. PhyloMaker: an R package that can generate very large phylogenies for vascular plants. Ecography. 2019.

8. Erickson DL, Jones FA, Swenson NG, Pei N, Bourg NA, Chen W, et al. Comparative evolutionary diversity and phylogenetic structure across multiple forest dynamics plots: a mega-phylogeny approach. Frontiers in genetics. 2014;5:358.

9. Hedges SB, Marin J, Suleski M, Paymer M, Kumar S. Tree of life reveals clock-like speciation and diversification. Mol Biol Evol. 2015;32(4):835-45.
